# Late-onset epileptic spasms: presentation, aetiology and outcome

**DOI:** 10.1093/braincomms/fcag224

**Published:** 2026-06-16

**Authors:** Sameer Dal, Emma Macdonald-Laurs, Simone Mandelstam, Rachel Kerr, Eunice K Chan, Kathryn Santamaria, Wirginia Maixner, Alison Wray, Colleen D’Arcy, Ramja Kokulan, Jacquie A Wrennall, Joseph Yuan-Mou Yang, Bonnie Alexander, Sila Genc, Sarah M Barton, Matthew Coleman, Paul J Lockhart, Richard Leventer, Jeremy L Freeman, Sarah E M Stephenson, Katherine B Howell, A Simon Harvey

**Affiliations:** Department of Neurology, The Royal Children's Hospital, Melbourne, Victoria 3052, Australia; Murdoch Children's Research Institute, Melbourne, Victoria 3052, Australia; Department of Neurology, The Royal Children's Hospital, Melbourne, Victoria 3052, Australia; Murdoch Children's Research Institute, Melbourne, Victoria 3052, Australia; Department of Paediatrics, The University of Melbourne, Melbourne, Victoria 3052, Australia; Department of Medical Imaging, The Royal Children's Hospital, Melbourne, Victoria 3052, Australia; Murdoch Children's Research Institute, Melbourne, Victoria 3052, Australia; Department of Neurology, The Royal Children's Hospital, Melbourne, Victoria 3052, Australia; Murdoch Children's Research Institute, Melbourne, Victoria 3052, Australia; Department of Paediatrics, The University of Melbourne, Melbourne, Victoria 3052, Australia; Department of Neurology, The Royal Children's Hospital, Melbourne, Victoria 3052, Australia; Department of Neurosurgery, The Royal Children's Hospital, Melbourne, Victoria 3052, Australia; Department of Neurosurgery, The Royal Children's Hospital, Melbourne, Victoria 3052, Australia; Department of Anatomical Pathology, The Royal Children's Hospital, Melbourne, Victoria 3052, Australia; Department of Neurology, The Royal Children's Hospital, Melbourne, Victoria 3052, Australia; Department of Psychology, The Royal Children's Hospital, Melbourne, Victoria 3052, Australia; Murdoch Children's Research Institute, Melbourne, Victoria 3052, Australia; Department of Paediatrics, The University of Melbourne, Melbourne, Victoria 3052, Australia; Department of Neurosurgery, The Royal Children's Hospital, Melbourne, Victoria 3052, Australia; Department of Neurosurgery, Neuroscience Advanced Clinical Imaging Service (NACIS), The Royal Children's Hospital, Melbourne 3052, Australia; Murdoch Children's Research Institute, Melbourne, Victoria 3052, Australia; Department of Neurosurgery, The Royal Children's Hospital, Melbourne, Victoria 3052, Australia; Murdoch Children's Research Institute, Melbourne, Victoria 3052, Australia; Department of Neurosurgery, The Royal Children's Hospital, Melbourne, Victoria 3052, Australia; Murdoch Children's Research Institute, Melbourne, Victoria 3052, Australia; Department of Neurosurgery, The Royal Children's Hospital, Melbourne, Victoria 3052, Australia; Murdoch Children's Research Institute, Melbourne, Victoria 3052, Australia; Department of Paediatrics, The University of Melbourne, Melbourne, Victoria 3052, Australia; Murdoch Children's Research Institute, Melbourne, Victoria 3052, Australia; Department of Paediatrics, The University of Melbourne, Melbourne, Victoria 3052, Australia; Department of Neurology, The Royal Children's Hospital, Melbourne, Victoria 3052, Australia; Murdoch Children's Research Institute, Melbourne, Victoria 3052, Australia; Department of Paediatrics, The University of Melbourne, Melbourne, Victoria 3052, Australia; Department of Neurology, The Royal Children's Hospital, Melbourne, Victoria 3052, Australia; Murdoch Children's Research Institute, Melbourne, Victoria 3052, Australia; Department of Neurology, The Royal Children's Hospital, Melbourne, Victoria 3052, Australia; Murdoch Children's Research Institute, Melbourne, Victoria 3052, Australia; Department of Paediatrics, The University of Melbourne, Melbourne, Victoria 3052, Australia; Department of Neurology, The Royal Children's Hospital, Melbourne, Victoria 3052, Australia; Murdoch Children's Research Institute, Melbourne, Victoria 3052, Australia; Department of Paediatrics, The University of Melbourne, Melbourne, Victoria 3052, Australia

**Keywords:** developmental and epileptic encephalopathy, infantile spasms, cognitive outcome, diagnostic delay, neuroimaging

## Abstract

Late-onset epileptic spasms (LOES) are epileptic spasms (ES) commencing after age 12 months, often misdiagnosed and having uncertain relationship to infantile spasms. Previous studies of mostly small LOES cohorts frequently reported ‘cryptogenic’ aetiologies. We studied the presentations, aetiologies, treatment responses and outcomes in a large LOES cohort, evaluated with modern neuroimaging and genomic testing. In this retrospective cohort study, 62 children with video-confirmed epileptic spasms, diagnosed between 2011 and 2021, were included. All had epileptiform activity on EEG, but none had hypsarrhythmia. Median age at epileptic spasms onset was 23 months (range 1–15 years) and median delay to diagnosis was 8 months (interquartile range: 3–15). Only 24% children were correctly diagnosed at presentation, common misdiagnoses being myoclonic epilepsies and non-epileptic phenomenon. Aetiology was identified in 95%. Structural-malformative aetiologies were present in 63% (most commonly focal cortical dysplasia and mild malformation of cortical development with oligodendroglial hyperplasia in epilepsy). Other aetiologies were structural-acquired in 13% (mostly postnatal stroke and CNS infections), genetic in 13% (mostly intragenic variants and chromosomopathies) and oncological in 6% (history of leukaemia, two with CNS involvement). Children with structural aetiologies often had normal development prior to onset of epileptic spasms, a later median age of epileptic spasms onset and were more likely to have asymmetric or subtle epileptic spasms and additional seizures prior to epileptic spasms. Epileptic spasms ceased in 29% children treated with prednisolone, most having genetic aetiologies and in 35% treated with vigabatrin, all having structural-malformative aetiologies. Apart from clobazam, which was effective in 17% of children, all other antiseizure medications, vagus nerve stimulation and ketogenic diet therapy were ineffective. Epileptic spasms ceased in 86% (18/21) of children who underwent epilepsy surgery. At median follow-up of 10.3 years, 53% children were free of all seizures and 76% were free of epileptic spasms. More children with unilateral structural-malformative aetiologies achieved seizure freedom than other aetiologies, most commonly following surgery but occasionally following treatment with vigabatrin or clobazam. Impairment of cognitive function or adaptive behaviour (formally assessed in 90%) was significantly more common in children with genetic than structural aetiologies, and in children with ongoing seizures than seizure freedom. Among children who underwent epilepsy surgery, 62% achieved average or low-average adaptive functioning or normal intellectual capacity. This study highlights the importance of prompt recognition of epileptic spasms in older children for improved seizure and developmental outcomes. Brain malformations and insults are the predominant causes of LOES, and when unilateral, respond best to epilepsy surgery.

## Teaser

Dal *et al*. report the clinical, EEG, imaging and genetic features of children with late-onset epileptic spasms. Despite having varied aetiologies, focal structural malformations were common. This study underscores the importance of high-resolution imaging and genetic testing in guiding treatment and prognostication.

## Graphical Abstract

**Graphical Abstract:**
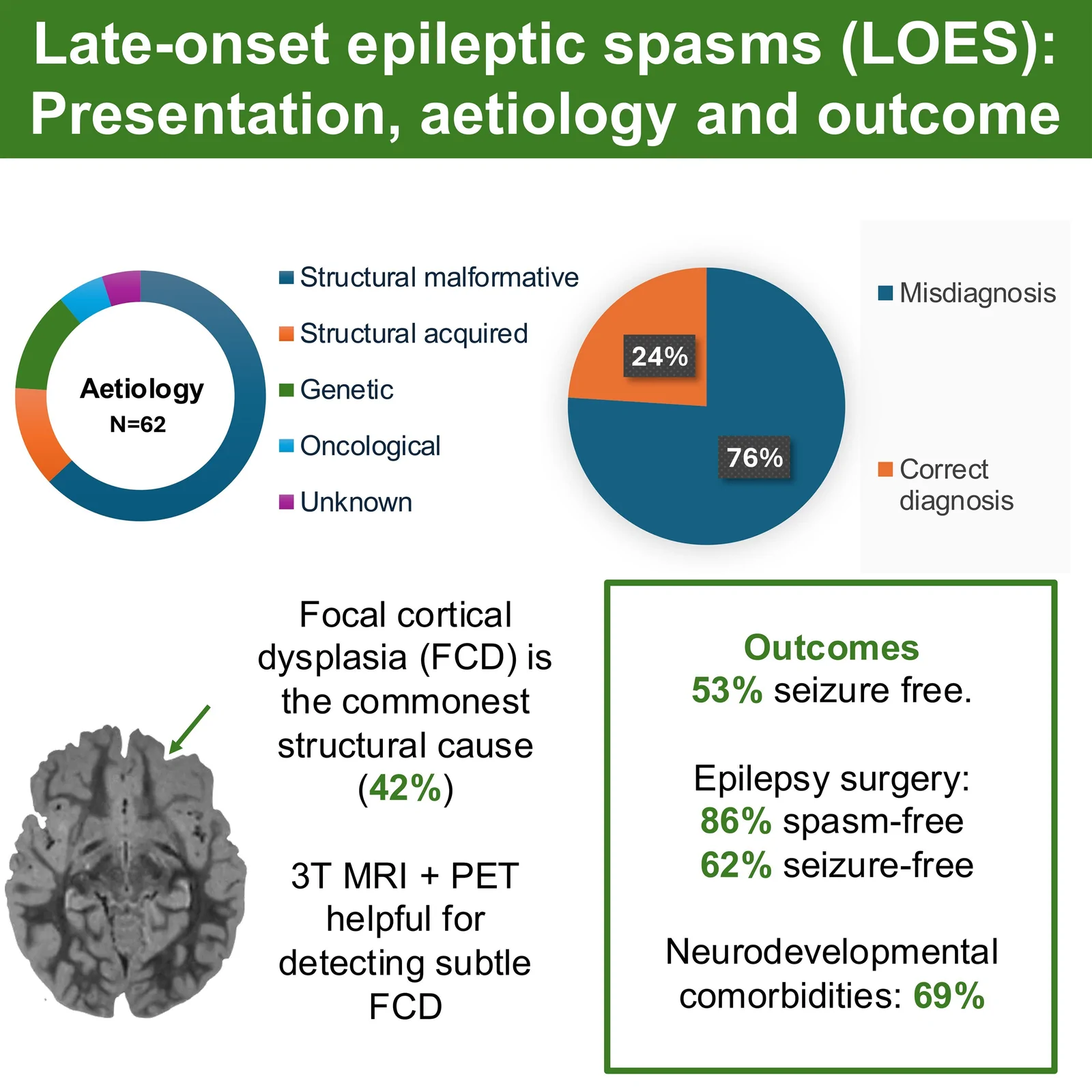

## Introduction

Infantile epileptic spasms syndrome (IESS) is the most common developmental and epileptic encephalopathy, with an estimated incidence of 2–5 per 10 000 births.^1-3^ Children with IESS present with clusters of epileptic spasms (ES), usually accompanied by developmental slowing or regression, and hypsarrhythmia on EEG. IESS has an age-related onset and offset, typically commencing between 3 and 9 months and then either ceasing or evolving into other epilepsies.

Approximately 2–8% of children with ES have onset after the first year of life (infancy).^4-6^ Onset of ES during the second year of life was only recently included within the ILAE definition of IESS,^2^ it previously having been considered within the entity of late-onset epileptic spasms (LOES).^7-10^ Why certain older children with epilepsy develop ES is poorly understood.^11^ It is unclear whether LOES represent the ‘tail-end’ of IESS, a distinct syndromic entity or a hybrid of IESS and Lennox–Gastaut syndrome (LGS).

Compared to children with IESS, children with LOES are reported to have a higher rate of structural or ‘cryptogenic’ causes, longer delay to diagnosis and non-hypsarrhythmic EEGs at presentation.^4-6^ Previously described LOES cohorts were mostly published prior to the era of advanced neuroimaging and genomic testing and had variable detail on long-term developmental and seizure outcomes. Diagnostic delays and aetiological uncertainty in LOES lead to delayed and ineffective treatment of ES with potential developmental impact.^7-9^

We describe a large group of children with LOES, focusing on presenting electroclinical features, aetiology, treatment response and long-term seizure and developmental outcomes. We used strict clinical, video and EEG criteria to diagnose ES and distinguish them from other seizure types. We highlight reasons for errors and delays in diagnosis of LOES and their underlying aetiology and consider their impact on treatment and outcome.

## Materials and methods

### Ethics approval and patient consent

The study was approved by The Royal Children's Hospital Human Research Ethics Committee (941874187). Informed consent was obtained prior to telephone interviews. Patient level data is reported in Supplementary Table 1. More detailed data is potentially available on request.

### Patient ascertainment and inclusion

Children with epilepsy treated at The Royal Children's Hospital, Melbourne, were screened for inclusion. Electronic medical record, EEG report and epilepsy case conference databases were searched during February 2022 and again in December 2023 using the terms: ‘spasms,’ ‘epileptic spasms,’ ‘infantile spasms,’ ‘refractory infantile spasms,’ ‘late-onset epileptic spasms,’ ‘symptomatic generalized epilepsy,’ and ‘West syndrome’. Video recordings by parents/caregivers and videos during EEG recordings of events were reviewed by at least three of four investigators (SD, KBH, EML, ASH) to confirm the diagnosis of ES. Medical records were reviewed to determine age of onset of ES.

Inclusion criteria were: (i) onset of ES after age 12 months (corrected if born <37 weeks gestation), (ii) onset of ES during the 11-year period 2011–2021, (iii) ES confirmed on review of caregiver provided home video or video-EEG recordings, and (iv) EEG with interictal epileptiform discharges (IEDs).

ES were defined as stereotyped movements occurring in clusters, including but not limited to sudden flexion and/or extension of the trunk and/or proximal limb muscles, subtle head nods, grimacing, altered facial expression and eye deviation. Movements had to be more sustained than myoclonus (>100 milliseconds) but briefer than a tonic seizure (<3 s). Clustering was defined as the occurrence of at least three ES within 60 s, though typically ES occurred more frequently and for a longer duration. Patients with ES immediately following a focal seizure were included, provided there was also a history of isolated ES in clusters.

IEDs included focal or generalized spike slow-wave discharges, sharp slow-wave discharges and paroxysmal fast activity. Where EEG was recorded during ES, the ictal EEG correlate had to show a diffuse di-phasic or tri-phasic slow wave with overriding fast or polyspike activity, with or without a preceding spike. However, ictal EEG was not mandatory for the diagnosis of ES, consistent with the approach adopted in other studies.^12,13^

Children whose suspected ES were shown on video or EEG to be myoclonic, atonic, myoclonic-atonic or tonic seizures were excluded. These and other seizure types occurring concomitantly with ES were allowed, except when ES were part of an LGS presentation, defined as multiple seizure types including tonic seizures and EEG showing generalized slow spike-wave and paroxysmal fast activity. Children presenting with only single, non-clustered ES were not included.

The initial database search returned 1092 children, reducing to 387 following removal of duplicates. Of these 387 children, 99 were screened as the initial search strategy indicated ES onset after age 12 months. 41 children were subsequently excluded following review of medical records and video recordings, reasons being age of ES onset under 12 months in five, seizures not ES in 32 and ES in the context of *de novo* LGS in four. When the search was repeated a year later, to capture potential delayed presentations and diagnoses, a further four children were included, yielding a total of 62 (39 male) (Supplementary Fig. 1).

### Clinical information

Clinical history, results of investigations and outcome were obtained from medical records and telephone interviews with parents or caregivers, conducted between June to December 2023. Information obtained included demographic details, perinatal, developmental and family histories and examination findings. A detailed seizure history was obtained, including confirmation of age at onset of ES and other seizures, age at diagnosis of ES, clinical features of ES, factors potentially contributing to delayed or erroneous diagnosis, clinical course, treatment response and outcome. Seizure outcome was rechecked and updated in February 2025. Response to anti-seizure medication (ASM) was defined as the cessation of ES for at least 12 months.

Results of EEG, neuroimaging, genetic and metabolic investigations were reviewed to establish aetiology. Aetiology was classified as structural (acquired or malformative), genetic (non-malformative), oncological or unknown.^1,14^

### Seizure videos and EEG review

Video recordings of ES were reviewed for all patients, being home videos (41%) and/or video-EEG (82%). All EEG recordings leading up to diagnosis of ES were reviewed by two investigators (SD, ASH) to document interictal EEG abnormalities and ictal EEG rhythms. EEG recordings from referring centres were sought, reports being used when not available. The most recent EEG recordings were reviewed to inform seizure outcome and syndrome evolution.

### Neuroimaging review

The most recent and highest quality brain MRI and ^18^F-FDG-PET (PET) scans were reviewed (unblinded) by two neurologists (SD, EML) and one neuroradiologist (SAM) investigator. MRI was done at 3T in 82% children with a previously described epilepsy protocol including volumetric T1 and FLAIR, and orthogonal T2 slices.^15^ PET was performed in 53% children, mostly on a Siemens Biograph scanner with simultaneous MRI acquisition. Focal cortical dysplasia (FCD) was suspected when there was increased cortical thickness, indistinct grey-white boundary and/or abnormal FLAIR/T2 cortical or subcortical signal on MRI, with or without accompanying hypometabolism on PET, imaging review sometimes aided by MRI-PET reformatting).^16^ Children with known acquired or genetic aetiologies did not typically undergo advanced neuroimaging.

### Genetics review

All children had some form of genetic testing; 45 had a chromosomal microarray and 24 had either an epilepsy gene panel or whole exome sequencing. The choice and sequence of tests varied among patients, and no standardized testing pathway was followed across the cohort. Ten of 21 children who underwent epilepsy surgery had genetic testing of resected tissue. All variants reported here are pathogenic or likely pathogenic.^17^

### Developmental assessments

Cognitive development preceding and following the onset of ES was assessed by two investigators (SD, KBH). Assessments were based on information recorded in medical records, formal developmental and cognitive assessments and parental interviews. Additionally, the Vineland Adaptive Behaviour Scale Vineland-3 parent/caregiver form was administered online during 2023 (76% completion).^18^ Fifty-six (90%) children had a formal developmental (Vineland-3) and/or cognitive assessment within a median of 15.5 months (interquartile range (IQR) 13–22.5) of last follow-up, the developmental outcomes for the remaining six children being determined by review of medical records. Cognitive assessments were conducted with the Wechsler Intelligence Scale for Children in 19 and the Wechsler Preschool and Primary Scale of Intelligence in 11. Associated neurodevelopmental and psychiatric comorbidities were recorded.

### Statistical analysis

Given the limited sample size, associations between categorical variables were tested with Fisher's exact test with statistical significance defined at *P* < .05. Despite this approach, some between-group differences may not have reached statistical significance.

## Results

### Seizure characteristics

**Figure 1:**
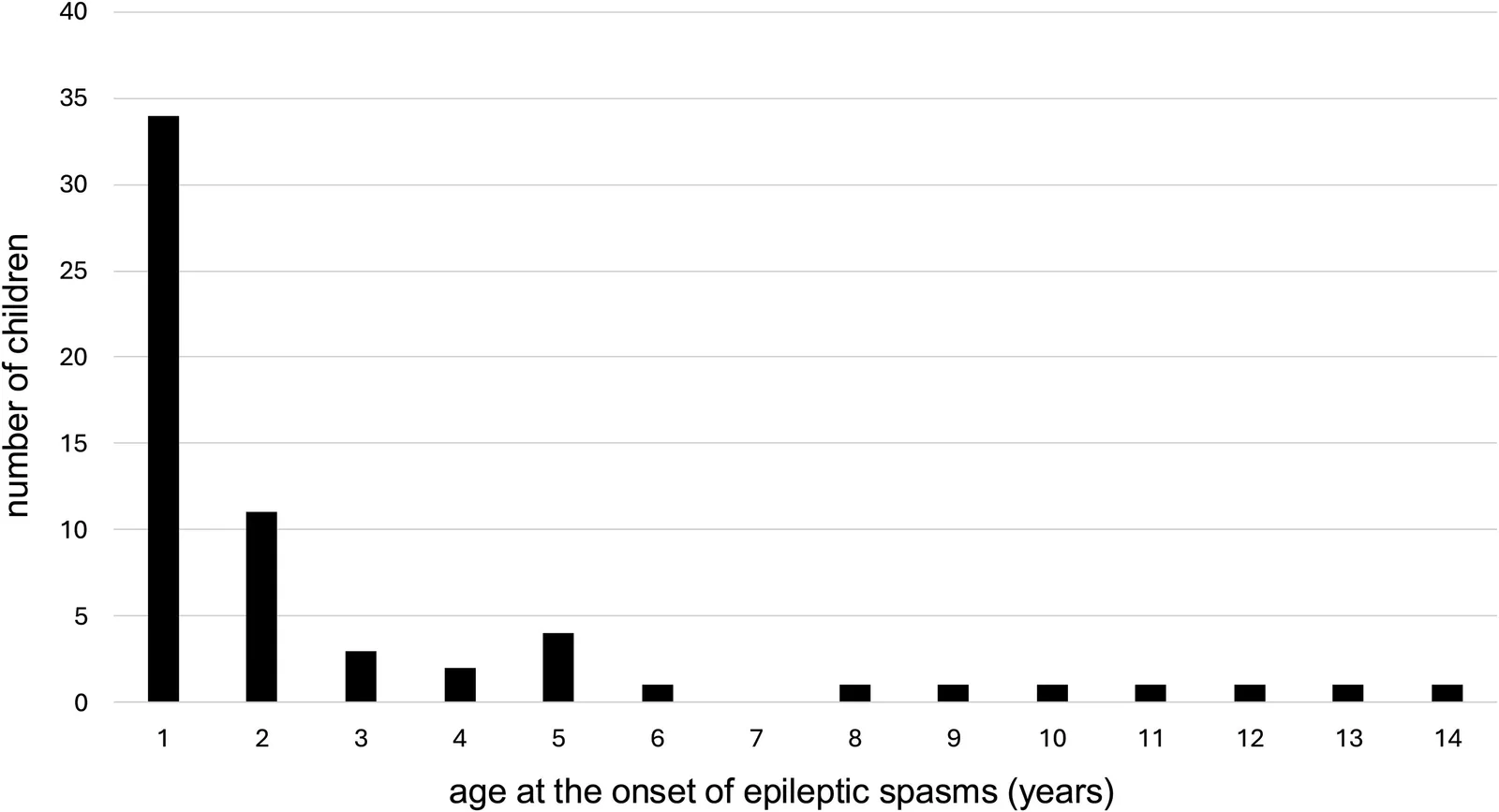
**Age at onset of late-onset epileptic spasms.** Histogram showing the age at onset of epileptic spasms in 62 children with late-onset epileptic spasms.

The median age at onset of ES in the 62 children was 23 (IQR 15–35.5) months, ranging from 12 months to 15 years (Fig. 1). The distribution was skewed to younger age at onset, with 34 children presenting with ES between age 12 and 24 months, 16 between 2 and 5 years and 12 after 5 years (Supplementary Table 1).

Clusters of ES usually occurred following waking from sleep, but in 22 (36%) children occurred at other times. Clusters often had a ‘crescendo-decrescendo’ tempo of ES, lasting 1–22 min.

In 40 (65%) children, ES manifested as episodes of upper limb abduction-extension, with truncal flexion in 37 and truncal extension in three. In the remaining 22 (35%) children, ES manifested as subtle movements including periodic chin contraction, lip pursing, jaw retropulsion or widening of the palpebral fissures. Four children with subtle ES had stereotyped mannerisms or automatisms after each EEG paroxysm, those being bringing one arm over their face, passively closing their eyes with one hand or clutching their groin with one hand. In 85% children, ES clusters were preceded by reduced interaction or cessation of activity, which persisted throughout the cluster, although usually with the impression of retained awareness. Two children with tuberous sclerosis complex (TSC) had focal motor seizures that were followed by ES, as well as separate clusters of isolated ES. Among the 40 children with trunk and limb movements in ES, asymmetry was present in 22 (55%), including subtle head and eye version and earlier or higher amplitude limb movements on one side.

Twenty-one (34%) children had other seizure types before the onset of ES. Eight children had acute symptomatic seizures, occurring in the context of CNS infection or stroke, 12–57 months prior to onset of ES. Thirteen children had focal seizures or focal to bilateral convulsive seizures, 3–25 months prior to onset of ES.

Nine (15%) children had other seizure types during the 4 weeks before or after onset of ES, four children having focal seizures and five having bilateral tonic–clonic seizures.

### EEG characteristics

IEDs were recorded during wakefulness in 55/62 (88%) children and sleep in 54/54. EEGs had >50% spike-wave during wake or sleep in 42 (67%) children. Focal slowing or focal IEDs were present in 46 (74%). No child had classical hypsarrhythmia or modified hypsarrhythmia.

**Figure 2:**
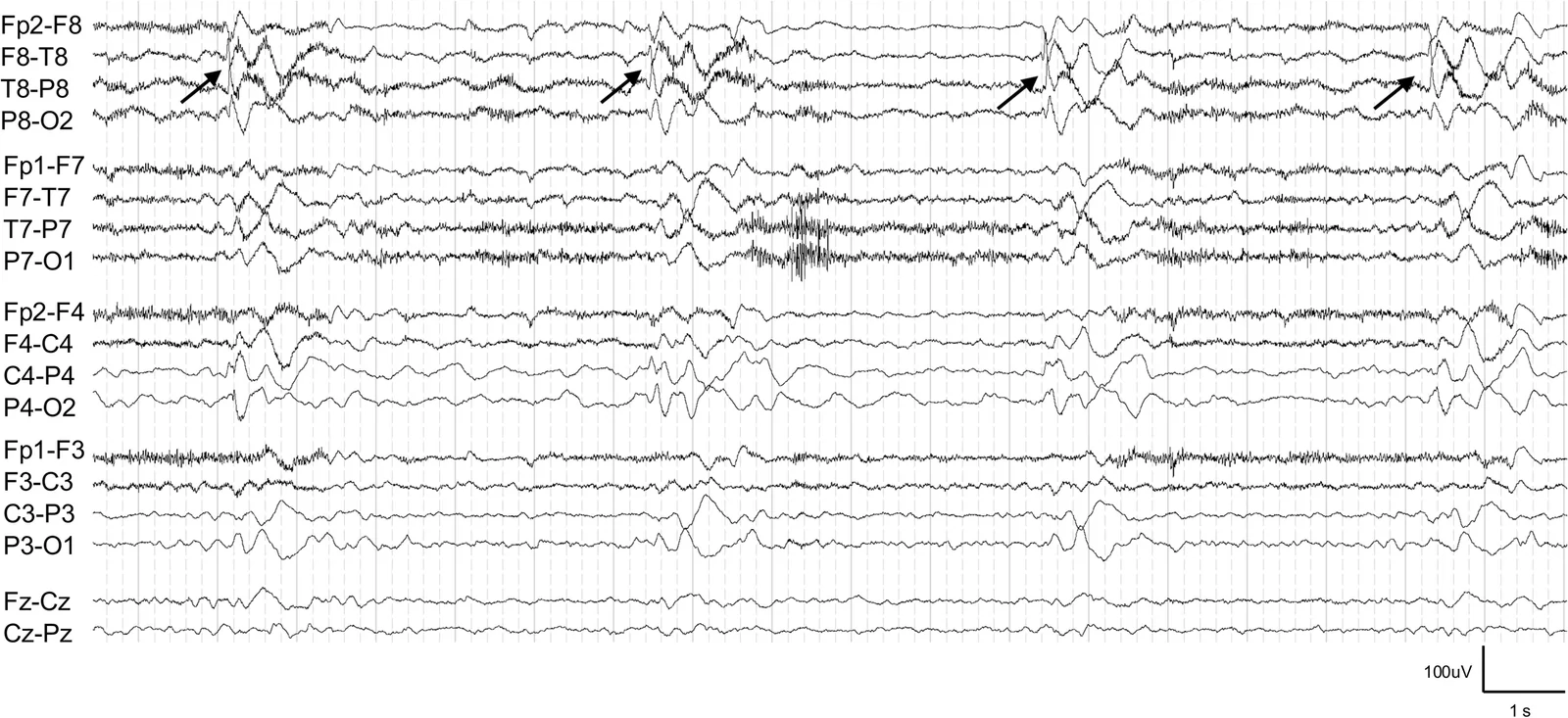
**Ictal electroencephalogram (EEG) recording of late-onset epileptic spasms.** Scalp EEG in a 29-month-old child with tuberous sclerosis complex and recent onset epileptic spasms. EEG channels are shown in a longitudinal bipolar montage in the vertical axis of the figure. The EEG signal is recorded in seconds (bold line) on the horizontal axis of the figure. EEG shows periodic ‘spasm complexes’ consisting of a diffuse slow wave with overriding fast activity, seen earlier and with higher amplitude on the right. Several spasm complexes are preceded by a ‘leading spike’ (arrow) over the right mid-temporal region. (High pass filter 70 Hertz (Hz), low pass filter 1 Hz, notch off, sensitivity 10 microvolt/millimetre).

During ES, recorded in 52 children, the ictal EEG showed broad, bi-phasic or tri-phasic, slow or sharp-slow wave complexes (‘spasm complexes’), with or without superimposed fast activity, preceding spike and after-coming attenuation (Fig. 2). These were seen periodically during clusters, with or without accompanying ES, often occurring with pseudo-normalization of interictal EEG. Focal features, including interhemispheric latency, voltage asymmetry or leading spikes were seen in 18/51 (35%) children with recorded ES (Fig. 2). These focal features often occurred early in the cluster, followed by more symmetric appearing spasm complexes. Rarely, there was activation of bilateral sharp-slow wave activity at the end of the cluster (*n* = 6). The two children with TSC and focal seizures preceding ES had focal ictal rhythms preceding spasm complexes with focal features.

### Diagnostic delays and errors

Median time from onset to diagnosis of ES was 8 months (IQR: 3–15). Twelve (19%) children encountered a diagnostic delay exceeding 12 months, with four misdiagnosed for 4–9 years. ES were diagnosed correctly at presentation in only 15 (24%) children. Correct diagnosis typically follows review of home video or video-EEG monitoring. Diagnostic delay was greater in children with ES onset after 2 years than in those with onset before 2 years (*P* = 0.03) (Table 1).

**Table 1:** Comparison of clinical, aetiological, EEG and outcome characteristics in children with late-onset epileptic spasms separated by spasm onset before or after age 2 years.^a^

	Age at onset of epileptic spasms		
	12–23 months (*n* = 34)	24 months or older (*n* = 28)	*P* value
**Clinical**			
Subtle epileptic spasms	10	12	Not significant
Asymmetric epileptic spasms	14	8	Not significant
Delay in diagnosis	22	25	0.03
Hypsarrhythmia	0	0	
**Aetiology**			
Structural malformative	23	16	Not significant
Structural acquired	3	5	
Genetic	7	1	Not significant
Oncological	0	4	0.03
Unknown	1	2	
**First-line treatment response**			
Prednisolone	7/19	0/5	Not significant
Vigabatrin	4/15	6/13	Not significant
**Outcome**			
Seizure free	22	11	Not significant
Normal development	7	10	Not significant
Evolution to Lennox–Gastaut syndrome	1	5	Not significant

^a^Because of the limited sample size, some between-group differences may not have reached statistical significance.

Many children received multiple misdiagnoses, including epilepsy with myoclonic or myoclonic-atonic seizures in 38, unspecified seizures of genetic generalized epilepsies in 26, absence seizures in eight, focal seizures in 24, tonic seizures in 10, anxiety or behavioural episodes in 16, tics in 10, ‘drop attacks’ in 10, parasomnias in eight, breath-holding spells in five and post-viral encephalopathy syndrome in one. Misdiagnoses were made while in the care of neurologists (72%), paediatricians (24%) and general practitioners (4%).

Delayed diagnoses were at least partly attributable to inaccurate interpretation of EEG in 10 children. In these children, bilateral broad sharp-slow waves were erroneously reported as generalized spike-waves and/or focal slowing or focal IEDs were not recognized within the dominant bilateral IEDs. In six children, clusters of subclinical spasm complexes were recorded during sleep but were not recognized.

### Aetiologies

**Figure 3:**
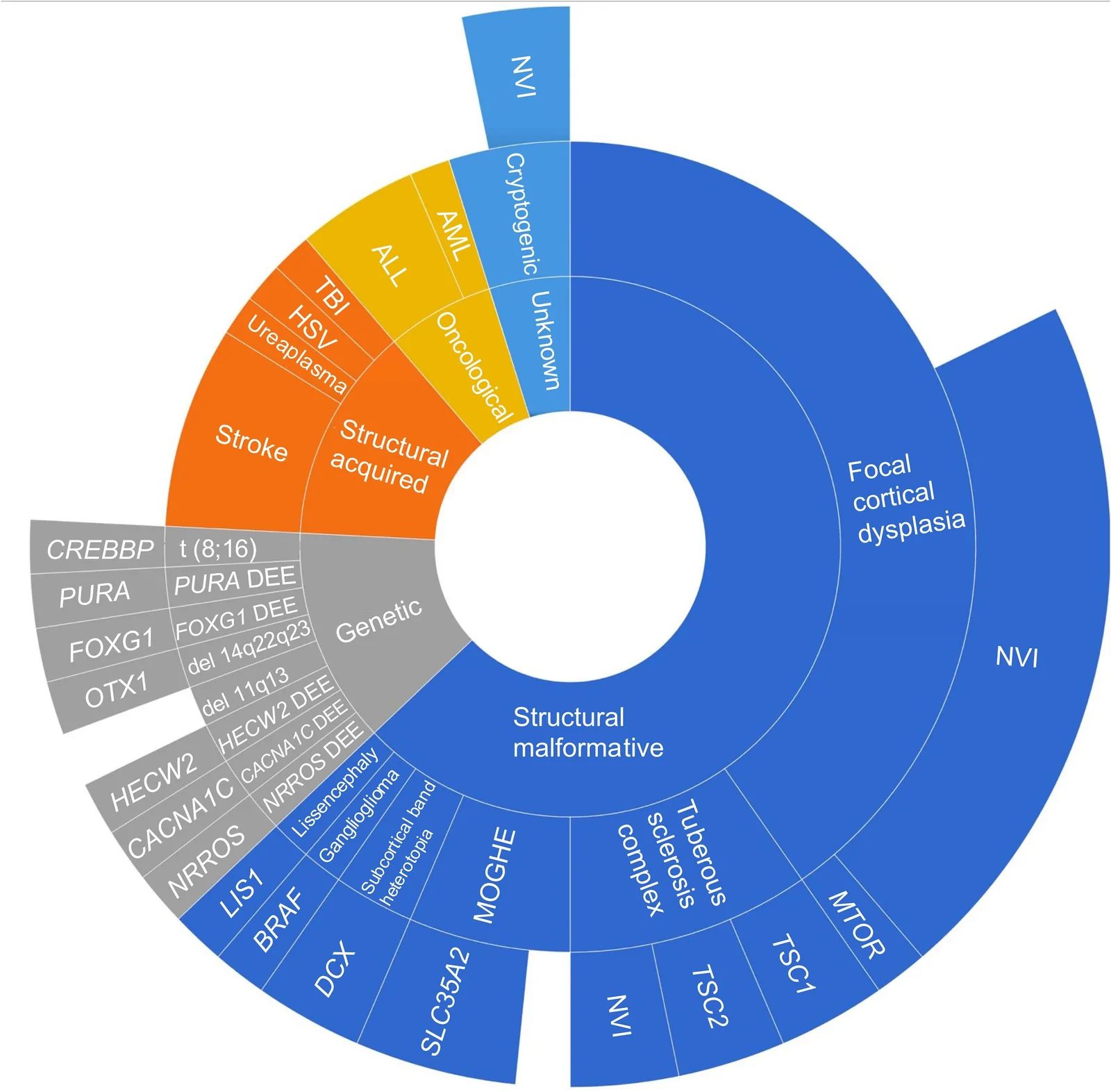
**Aetiology of late-onset epileptic spasms.** Starburst chart showing the aetiological categories (inner circle), the specific aetiologies (middle circle) and the underlying genetic variants (outer circle) in 62 children with late-onset epileptic spasms. Abbreviations: MOGHE (mild malformation of cortical development with oligodendroglial hyperplasia), *NRROS* (negative regulator of reactive oxygen species), *CACNA1C* (calcium voltage-gated channel subunit alpha1 C), *HECW2* (HECT, C2 and WW domain containing E3 ubiquitin protein ligase 2), *FOXG1*(forkhead-box G1), PURA (purine-rich element-binding protein A), HSV (herpes simplex virus), TBI (traumatic brain Injury), HHV6 (human herpes virus 6), NVI (no variant detected), mTOR (mechanistic target of rapamycin), *TSC* 1 & 2 (tuberous sclerosis complex 1 & 2), *SLC35A2* (solute career family 35 A2), *DCX* (doublecortin), *BRAF* (B-raf protooncogene), *LIS1* (lissencephaly 1), *OTX1* (orthodenticle homeobox 1) and *CREBBP* (c-adenosine monophosphate response element-binding protein).

An aetiology for LOES was determined in 59 (95%) children. Aetiologies were structural-malformative in 39 (63%) children, structural-acquired in eight (13%), genetic in eight (13%), oncological in four (6%) and unknown in three (5%) (Fig. 3). Structural abnormalities were unilateral in 30, predominantly unilateral in six and bilateral in 11.

**Figure 4:**
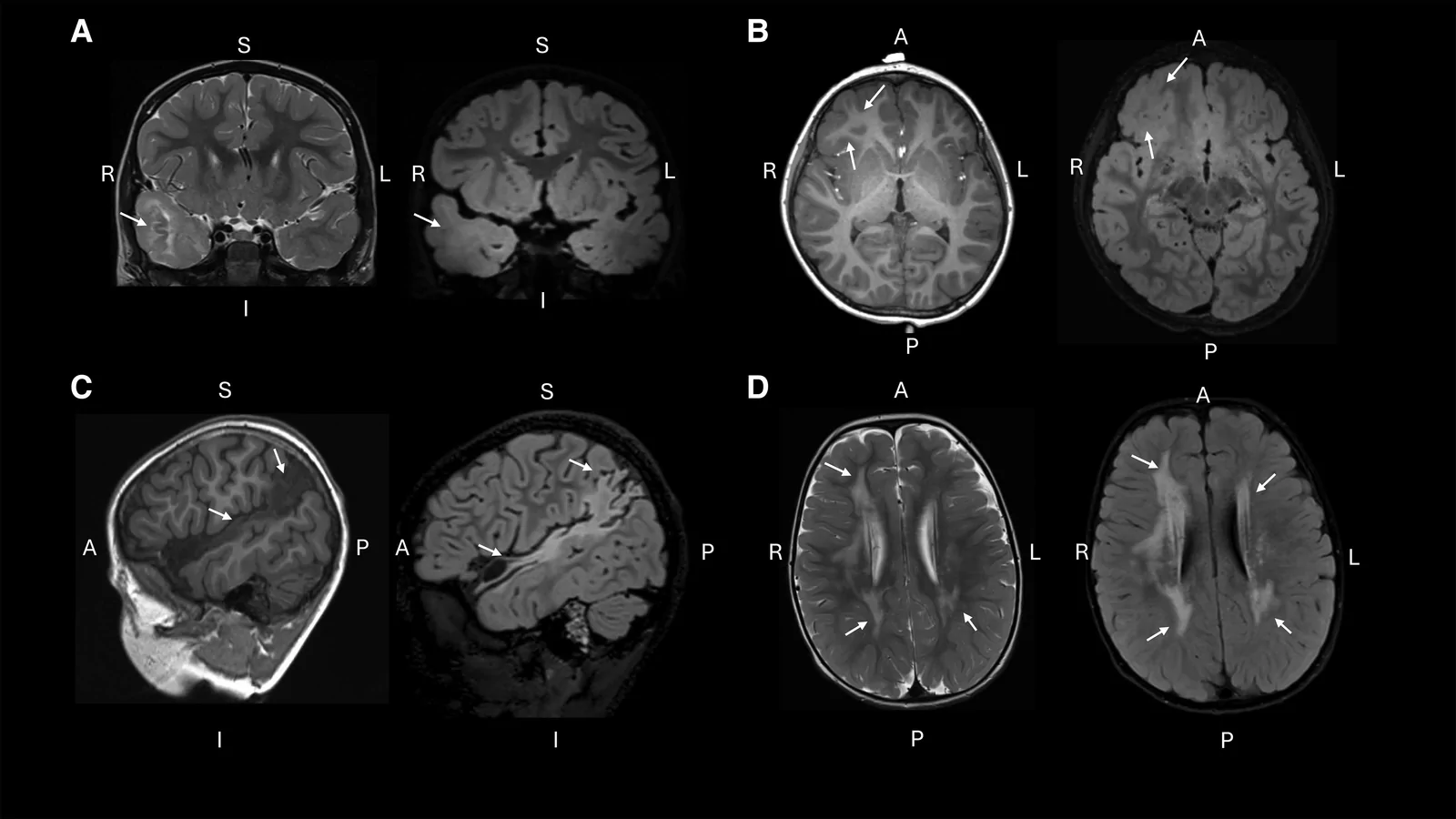
**Examples of MRI findings in late-onset epileptic spasms (all images are on radiological view and abnormalities are arrowed).** (**A**) Coronal T2-weighted and double inversion recovery (DIR) MRI of a male child with the onset of epileptic spasms at the age 20 months, showing a right temporal ganglioglioma (confirmed on histopathology examination). (**B**) Axial T1-weighted and DIR MRI of a male child with ES onset at 27 months of age, showing mild malformation of cortical development with oligodendroglial hyperplasia (*SLC35A2* (Solute Carrier Family 35 Member) pathogenic variant on genetic testing of resected tissue) involving the right orbitofrontal cortex. (**C**) Sagittal T1-weighted and DIR MRI of a male with epileptic spasm onset at 28 months of age showing encephalomalacia in the left superior temporal, supramarginal and angular gyri, secondary to venous infarction at age 16 months. (**D**) Axial T2-weighted and fluid-attenuated inversion recovery MRI of a female with a history of acute myeloid leukaemia and onset of epileptic spasms at 11 years of age, showing extensive white matter signal abnormality and volume loss, consistent with methotrexate-induced chronic leukoencephalopathy. S = superior, I = inferior, R = right, L = left, A = anterior, *P* = posterior.

Structural malformative aetiologies based on MRI were suspected FCD in 26 (67%) children, suspected mild malformation of cortical development with oligodendroglial hyperplasia in epilepsy (MOGHE) in three, tumour in one, TSC in six, subcortical band heterotopia in two and lissencephaly in one. Histology through epilepsy surgery confirmed FCDI in five, FCDII in five, ganglioglioma in one (Fig. 4A), MOGHE in two and TSC in three; two patients had non-diagnostic pathology. Somatic variants were identified in brain tissue of four operated patients (*SLC35A2* in two, *BRAF1* in one and *MTOR* in one). Germline variants identified were *TSC1* in two, *TSC2* in two, *LIS1* in one and *DCX* in two (Fig. 3). FCD and MOGHE (Fig. 4B) involved the frontal lobe in 17/29 children, particularly the frontal operculum orbitofrontal and anterior cingulate regions. Of these, 13 were on the right and 4 on the left.

Structural-acquired aetiologies were stroke in five, CNS infections in two (herpes simplex virus (HSV) and ureaplasma) and traumatic brain injury in one. All had acute symptomatic seizures prior to the onset of ES. Strokes were arterial in four and venous in one, being left-sided in four and having variable lobar involvement (Fig. 4C). These brain insults occurred at age one week to 73 months, 23–56 months prior to the onset of ES.

Eight children had confirmed genetic (non-malformative) aetiologies. Five had an intragenic variant (*CACNA1C*, *FOXG1*, *HECW2*, *NRROS* and *PURA*), two had chromosomal microdeletions and one had unbalanced translocation of chromosomes 8 and 16. All but the *PURA* and *CACNA1C* variants were known prior to the onset of ES, identified during investigation of developmental delay. MRIs showed non-specific abnormalities not warranting inclusion in the structural-malformative group, including global atrophy in three patients, bilateral dysmorphic hippocampi in one and hippocampal sclerosis in one. Six children had a first-degree relative with a history of febrile seizures and three had a history of epilepsy, these being the only children with LOES and a family history of seizures.

Three children had acute lymphoblastic leukaemia (ALL) and one had acute myeloid leukaemia (AML) prior to the onset of ES. All four children received chemotherapy, including intrathecal methotrexate in three. One child had CNS involvement at presentation. One child had CNS relapse, subsequently receiving neuroaxis irradiation and bone marrow transplant, complicated by HHV6 infection and posttransplant acute limbic encephalitis (PALE). ES commenced 2–11 years after commencement of induction therapy. MRI showed extensive bilateral white matter changes in two (Fig. 4D), signal abnormality in the amygdala and loss of internal architecture of the anterior hippocampus in the child with PALE, and no abnormalities in one. All had subtle manifestations of ES, such as clusters of eyes rolling or head nods while awake or repeated eye-opening during sleep that were shown on video-EEG to be ES, not tonic seizures.

In three children, the aetiology for LOES was not determined despite 3T MRI, PET, exome sequencing, chromosomal microarray, testing for common mitochondrial variants and metabolic investigations.

Compared to other aetiologies, children with structural aetiologies were more likely to have normal development prior to onset of ES (24/47 versus 3/15, *P* = 0.04), later median age at onset of ES (33 months versus 16 months, *P* = 0.03), asymmetric ES (21/47 versus 1/15, *P* = 0.01), subtle ES (13/47 versus 9/15, *P*  *=* 0.03) and other seizure types (20/47 versus 1/15, *P*  *=* 0.01). Oncological aetiologies were significantly more common in children with ES onset after age 24 months. There was no significant difference in the proportion of other aetiologies in children with ES onset before versus after age 24 months (Table 1).

### Treatment trials and epileptic spasm response

**Figure 5:**
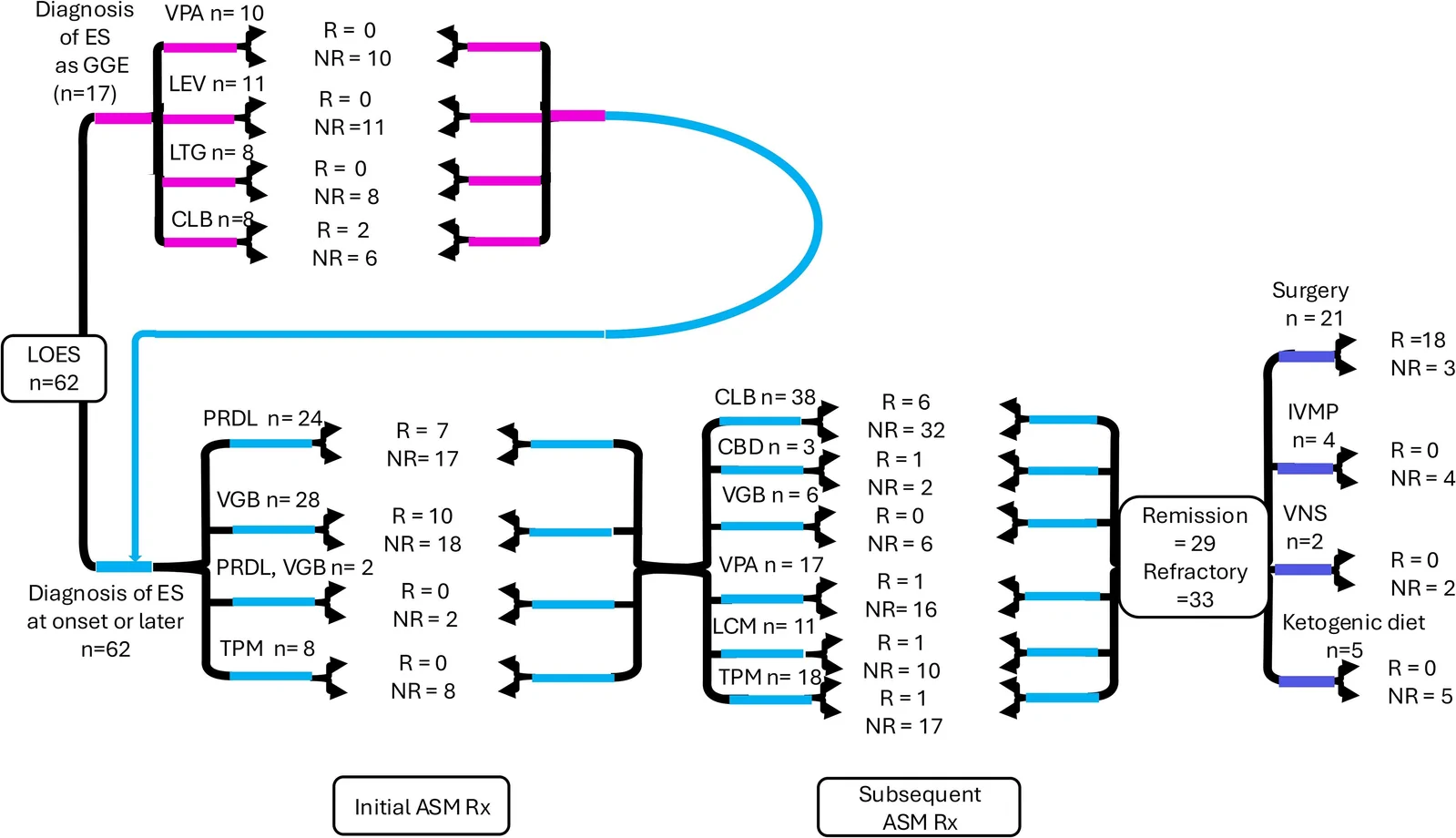
**Treatment of late-onset epileptic spasms.** Cumulative treatment regimens for 62 children with late-onset epileptic spasms, categorized by correct and incorrect initial diagnoses. Treatment response was defined as the cessation of epileptic spasms for 12 months or longer. The response was attributed to a particular medical or surgical treatment when the cessation was observed contemporaneously with the introduction of that therapy. ASM = anti-seizure medication, CBD = cannabidiol, CLB = clobazam, GGE = genetic generalized epilepsy, IVMP = intravenous methylprednisolone, LCM = lacosamide, LEV = levetiracetam, LOES = late-onset epileptic spasms, LTG = lamotrigine, NR = non-responder, PRDL = prednisolone, R = responder, TPM = topiramate, VPA = sodium valproate, VGB = vigabatrin, VNS = vagus nerve stimulation.

Seventeen children were treated with ASM not specific for ES, including levetiracetam, valproate, lamotrigine and clobazam (Fig. 5). Such ASM treatment was used for a median of 13.5 (IQR 4.5–27) months, the longest duration being nine years. Only 2/17 children responded to treatment (cessation of ES for 12 months or longer), both treated with clobazam.

When ES were correctly diagnosed, either at presentation or following misdiagnosis, initial treatment was high-dose oral prednisolone^12^ in 24 children, vigabatrin in 28, combined vigabatrin and prednisolone in two and topiramate in eight. Prednisolone ceased ES in 7/24 (29%) treated children, all responders having onset of ES during the second year of life (13–16 months). Only three of the prednisolone-responsive children had structural aetiologies, the remainder had genetic aetiologies. Vigabatrin was effective in ceasing ES in 10/28 (36%) treated children, all having structural malformative aetiologies. There was no significant difference in response to treatment with prednisolone or vigabatrin in children with ES onset before versus after age 24 months (Table 1). The combination of vigabatrin and prednisolone was not effective in ceasing ES in two children with unknown aetiologies.

When ES persisted or relapsed after correct diagnosis and initial treatment, various other ASMs were trialled as monotherapy or adjunctive treatment (Fig. 5). Response to ASMs such as topiramate, lacosamide, valproate and cannabidiol was poor, but 6 out of 32 (19%) children responded to clobazam.

No response was seen with ketogenic diet therapy in five children, vagus nerve stimulation in two and pulse intravenous methylprednisolone in four. Epilepsy surgery was performed in 21 children at a median age of 54 (IQR: 31–76) months and a median of 4 years after the onset of ES. Epilepsy surgeries were corticectomies or lobectomies in 16 children, tubectomies in three and hemispherotomies in two. Eighteen (86%) operated children had cessation of ES, with a median follow-up of 8 (IQR 5–11) years.

### Seizure outcome

At last follow-up, at age 9 (IQR: 7.5–13.3) years and 7 (IQR: 5–11) years after ES onset, 33 children (53%) had been free from all seizures for at least 12 months. Thirteen of these children, all with structural aetiologies, had undergone epilepsy surgery and were not on ASM. Fifteen of the remaining 20 children without seizures also had structural aetiologies, with seizure remission on vigabatrin (33%), clobazam (27%) or combinations of these and other ASM. Among the other five children without seizures, two on ASM, four had genetic and one had oncological aetiologies.

Twenty-nine children had ongoing seizures, on one (28%) or several (72%) ASM. Three children had only ES, 12 had ES and other seizures and 14 had other seizures (focal or focal to bilateral tonic clonic) only. Eleven children had tonic seizures but only six had evolved to classical LGS as defined, one or two from each aetiological group. All children with unknown, most with oncological and about half with genetic aetiologies (10/15 in total) had ongoing seizures, in comparison to 19/47 children with structural aetiologies (*NS*). However, children with unilateral structural-malformative aetiologies had better seizure outcomes when compared to all other aetiologies (21/30 versus 9/23, *P* = 0.029), reflecting their surgical interventions in most cases but seizure remission on ASM in some.

### Developmental outcomes

Developmental delay prior to onset of ES was present in 35 (56%) children, being more common in children with genetic compared to other aetiologies (8/8 versus 27/54, *P* = 0.008). All children with genetic aetiologies had global developmental delay prior to onset of ES. In contrast, normal prior development or isolated language or motor delay was mainly present in children with structural aetiologies. Developmental slowing or regression after onset of ES occurred in 21 (34%) children.

At follow-up, only 17 (27%) children were functioning in the average range. Of the 30 children who underwent IQ-based assessments, eight had mild intellectual disability (ID), eight had moderate and 11 had severe. Of the 47 children whose carers completed the Vineland-3 at follow-up, the adaptive behaviour composite (ABC) score indicated 11 children had average or low-average functioning, 29 had mild-moderately impaired functioning and seven had severely impaired functioning. These VABS and IQ-based assessments were done at a median age of 8 (5–11.5) years. Of the six children who had neither VABS nor IQ-based assessments, three had significant delays and three were within the normal range, based on review of notes, parental interviews and schooling.

Functioning within the impaired range was more common in children with genetic than structural aetiologies (8/8 versus 29/47, *P* = .04). Impairment was also more common in children with ongoing seizures at last follow-up compared to those without (18/33 versus 27/29, *P* = .0007). Developmental outcome did not differ with the onset of ES before or after age two years. The poorest developmental outcomes occurred in children with genetic, unknown and oncological aetiologies, most of whom had ongoing seizures. Among children who underwent epilepsy surgery, 13/21 (62%) had either average or low-average adaptive functioning or normal intellectual capacity.

Sixty-nine percent of children had neurodevelopmental comorbidities, including 32% with autism, 21% with ADHD and 62% with anxiety. Thirty-two children attended mainstream schools, with eight receiving additional support or an adjusted curriculum. Twenty-six (42%) children attended special schools. Four children were not yet enrolled in school.

## Discussion

We report a large cohort of children with LOES in whom the diagnosis was robust, based on strict clinical and EEG criteria with video confirmation. In most children, ES were the only or predominant seizure type at onset and during the course of their epilepsy. We excluded children with myoclonic and tonic seizures masquerading as ES, and children in whom ES were part of a LGS presentation, differentiating our approach from previous studies.^5,9,19^ As such, we ascertained a ‘pure cohort’ of children with LOES and examined clinical, aetiological and outcome features, with the aim of improving recognition and management.

### Electroclinical features of late-onset epileptic spasms

In our cohort, ES had diverse manifestations, with many children exhibiting asymmetric or subtle ES, unlike the predominant symmetrical flexor ES in infants. Children with structural aetiologies were more likely to have asymmetric or subtle ES and often had other seizures prior to the onset of ES. Regardless of the symmetry or subtlety, clustering and periodicity of ES were striking features in all, being a justifiable inclusion criterion to help distinguish ES from single tonic and myoclonic seizures, which potentially were present in some prior studies of LOES.^5,9,19^

Many researchers have emphasized the paucity of hypsarrhythmia and its variants in the interictal EEG of children with LOES.^7,8,10^ In our cohort, only one child had modified hypsarrhythmia. Instead, preservation of some normal background activity and intermittent rather than continuous IEDs were common EEG features. The ictal EEG correlate of ES in LOES was similar to that in IESS.

The established first-line treatments for IESS are adrenocorticotropic hormone, prednisolone and vigabatrin.^12,13^ However, their efficacy in LOES is less established than in IESS. In one study of 42 patients with LOES, there were only 18 treatment responders.^9^ In another study, only 2/22 treated with vigabatrin and 8/21 treated with steroids responded.^5^

Although our study was not intended to establish treatment guidelines, our findings suggest that vigabatrin and clobazam are moderately effective for LOES in children with a structural aetiology and prednisolone was slightly better than vigabatrin in children with a genetic aetiology, the latter suggested in another study.^9^ The efficacy of vigabatrin in structural-malformative aetiologies of ES is recognized.^20,21^ In older children with LOES, first-line treatment is variable in our centre, with vigabatrin and clobazam used more commonly than prednisolone, while aetiology is being investigated.

At follow-up, half (54%) of our cohort achieved seizure freedom, compared to a third in long-term studies of IESS.^22-27^ Among those with persistent seizures at last follow-up, approximately half had ES exclusively, while the remainder had other seizure types, with or without ES. Notably, only six children (10%) progressed to LGS, with no aetiological or age at onset association. Evolution to LGS is seemingly less frequent in children with LOES (3–10%) compared to those with IESS (approximately 30%),^2,4-6,9^ our and other studies having sufficient duration of follow-up. Children with persistent seizures at last follow-up demonstrated no significant difference between aetiological subgroups, though those with unilateral structural-malformative aetiologies specifically did significantly better. A study of LOES in which structural aetiologies predominated (82%) reported seizure outcomes similar to ours, with 53% of children achieving seizure freedom at last follow-up and none evolving to LGS.^8^

Previous studies report moderate to severe developmental delay in 60–80% of patients with LOES.^4-6,9^ In our study, 44% of children had normal development prior to the onset of ES. However, at follow-up, only 27% had normal development, with many children diagnosed with neurodevelopmental disorders, including ID. Poor developmental outcomes were seen more frequently in children who did not achieve seizure control with ASMs or surgery. These findings align with previous studies in children with LOES^9,10^ and IESS,^2,22-27^ emphasizing the potential impact of delayed diagnosis and treatment, poor initial response to therapy, and persistent seizures at follow-up, all of which are associated with unfavourable long-term cognitive outcomes.

### Aetiologies of late-onset epileptic spasms and treatment implications

Overall, aetiology was identified in 95% of our children with LOES, contrasting reports from previous decades in which the aetiology was categorized as cryptogenic in many.^5,7^ The near elimination of cryptogenic aetiologies in our series can be attributed to high-resolution MRI with PET and genetic testing.

Structural-malformative aetiologies were the commonest, with FCD and MOGHE comprising almost half of cases (47%).^6,7,10^ FCD was more commonly located in the frontal lobe, more frequently on the right and most commonly in the orbitofrontal and opercular regions. Cerebral lesions in the occipital and centro-temporal regions are associated with earlier onset of ES, while frontal lesions are associated with later onset, presumably reflecting the later maturation of the frontal cortex and explaining the predominance of frontal FCD in LOES.^28,29^ MRI in children with LOES and no apparent aetiology should therefore be scrutinized for FCD and MOGHE in the frontal lobes, particularly the basal and opercular regions. Many of the operated FCD were multilobar and had type 1 histopathology, such as malformations of cortical development with dyslamination being prominent in epilepsy surgical series of infants and young children with epileptic spasms.^30,31^ We attribute the high proportion of focal malformations in our cohort to the use of combined PET and 3T MRI with expert review.^16^

Acquired structural aetiologies included infections such as HSV encephalitis and stroke, commonly associated with acute symptomatic seizures. The late onset of ES is not simply attributable to later life insults, as half had cerebral insults during the perinatal period. In a series of 22 children with HSV encephalitis, 14 developed ES, commencing after age 1 year in most children. The combination of temporal pole, insular and hippocampal involvement was most strongly associated with the occurrence of ES, mirroring the radiological findings seen in our patient.^32^ In two case reports of ES following HSV encephalitis, commencing at age 16 months, brain injury involved both temporal lobes.^33,34^ It is unknown why ES associated with HSV encephalitis tends to have a late onset.

Epilepsy surgery was undertaken in 34% of our cohort, reflecting the high proportion of unilateral structural aetiologies and the ability of high-quality MRI and PET to identify subtle abnormalities. Most operated children achieved cessation of ES, with eight having persistent seizures at the last follow-up. Seizure freedom following surgery was strongly associated with favourable cognitive outcome. A previous study of LOES reported comparable findings.^8^ Comparatively favourable seizure and developmental outcomes in children with unilateral malformations who underwent surgery justify the search for such lesions on neuroimaging, especially in children with no historical antecedent, normal genetic testing, normal prior development, asymmetries of ES or focal features on EEG. The better developmental outcomes in children with unilateral malformations also suggest that development is likely influenced by the underlying aetiology and the effects of seizures. Unilateral structural-malformative aetiologies typically involve only localized brain areas, offering the potential to eradicate seizures and promote development.

A variety of chromosomal and single-gene disorders made up the genetic aetiologies, comprising 13% of children with LOES. Others have shown associations of LOES with *MECP2* duplication and Pallister–Killian syndromes,^35-37^ not represented in our cohort. A retrospective study examining the epilepsy phenotype in *MECP2* duplication syndrome found that 50% of patients showed electroclinical features of LOES, with a median onset age of 6 years.^33^ Notably, none of these cases progressed to LGS. Another study involving eight epilepsy patients with *MECP2* duplication syndrome reported that six experienced seizures characterized by recurrent head drops or loss of tone, often upon awakening; however, the absence of ictal recordings precluded confirmation of LOES.^36^ Moreover, other reports highlight that LOES can be an infrequent feature of several neurodevelopmental genes where epilepsy (but not LOES) is a common feature (*POLR2A, ADA2, WDR45, GRIA2, CASK, CDKL5, HEXA* and *GFAP*).^9^ While LOES due to genetic aetiologies commonly responded to first-line therapy with prednisolone, persistent seizures at follow-up were present in 38% and the majority had developmental delay.

In our cohort, four children with leukaemia developed ES, only two with CNS involvement, several years after their diagnosis and treatment. In the absence of an identifiable mechanism and evidence of neocortical injury, these children were not included in the structural-acquired aetiological group and the mechanism of their epilepsy is unclear. None had acute symptomatic seizures or posterior reversible encephalopathy syndrome. Neurotoxicity from intrathecal methotrexate and cranial radiotherapy may be contributory, with symptomatic focal and generalized epilepsy syndromes reported years post-remission.^38,39^ HHV6-related PALE was likely contributory in one patient. Children with drug-resistant epilepsy, a history of cancer treatment and no cerebral injury on MRI should undergo video-EEG monitoring to assess for LOES and associated epileptic encephalopathy.

In three children, the aetiology was unknown. The aetiology in these children could conceivably be genetic, due to deep intronic variants, low-level mosaicism, structural variants or epigenetic influences on regulatory genomic regions. Alternatively, they could have an occult cortical malformation, especially as none had developmental delay prior to the onset of ES.

### Distinct electroclinical syndrome or IESS continuum?

LOES is a clinical entity with both overlapping and distinct features when compared to IESS. While clustering of ES is a common feature of both IESS and LOES, LOES are more often asymmetric or subtle (24–68% versus 0.6–20% in IESS),^2,6,7,10^ and less strongly associated with sleep states. Other distinct features include the higher frequency of structural aetiologies (53–82% versus 38–50% in IESS),^6-8,40-42^ absence of hypsarrhythmia (versus ∼80% in IESS),^6-9,43^ less frequent response to steroids (10–24% versus 70–76% in IESS) and less frequent progression to LGS (3–10% versus ∼30% in IESS). Seizure outcomes are slightly better in LOES compared to IESS, yet developmental outcomes remain poor in both.

ES at any age likely results from a common and specific cortical-subcortical interaction, reflecting a shared ‘secondary seizure network’.^44^ The involvement of subcortical and brainstem centres in ES is suggested by PET findings, motor features of ES (resembling Moro reflex) and association with the sleep-wake cycle.^45,46^ The cortex being the source of ES and the subcortical-brainstem structures being secondary are suggested by resolution of ES following cortical resection.^16,31,47,48^ While ES results from a range of aetiologies, the type and location of these may contribute to ES presenting at different ages, potentially relating to network maturation.

It has been proposed that LOES represents a distinct entity within the group of developmental and epileptic encephalopathies, alongside syndromes such as early infantile developmental and epileptic encephalopathy (EIDEE), IESS and LGS.^5,49^ Various nomenclatures have been suggested for LOES, with ‘late-onset infantile epileptic encephalopathy’ (LIEE) being the most recent.^19^ This term implies that LOES represents late presentations of IESS, but the differences in electroclinical manifestations, aetiologies, treatment response and evolution suggest otherwise. Distinction of LOES from LGS was apparent in our study from electroclinical features, such as patients being excluded at presentation. Additionally, evolution to LGS was infrequent in our and other LOES cohorts.

We believe the weight of evidence supports LOES being considered an epileptic syndrome distinct from IESS and LGS, albeit with phenotypic overlap and shared pathogenic mechanisms for the ES. The extension of IESS to include ES onset into the second year of life helps to capture the majority of children with LOES, but clinical differences like absence of hypsarrhythmia need to be remembered.

### Diagnostic challenges and their implications

Our study highlights the challenges with correct and timely diagnosis of LOES, with a significant proportion of children being misdiagnosed for extended periods, leading to delays in appropriate drug and surgical treatments and possible impacts on their development. Recurring themes in misdiagnoses were impressions of ES occurring only in infants, overreliance of EEG reporting neurologists on the presence of hypsarrhythmia for diagnosing ES, missing focal interictal EEG findings and misinterpretation of head drops and bilaterally synchronous IEDs as myoclonic epilepsy. Home video recordings and video-EEG monitoring were pivotal in achieving accurate diagnosis, the quality of the epileptic movements and their periodic occurrence being key. High-quality MRI and advanced post-processing led to greater detection of subtle structural abnormalities, allowing more children to be identified as surgical candidates and offering them the best chance at achieving seizure freedom.

Greater awareness among neurologists and paediatricians about the potential onset of ES in later childhood, the sometimes subtle and asymmetric clinical and EEG features of LOES, and the importance of genetic testing and high-quality neuroimaging and review are needed. Recognition of LOES as a distinct epileptic syndrome or de-emphasizing infancy by removing it from IESS (i.e. syndrome of epileptic spasms), would potentially improve diagnosis, treatment and outcome of LOES.

## Supplementary Material

fcag224_Supplementary_Data

## Data Availability

The data that support the findings of this study are available from the corresponding author upon reasonable request.
