# Supplementary material for: Late-onset epileptic spasms: presentation, aetiology and outcome
Source: Brain Commun. 2026 Jun 16;8(4):fcag224. doi: 10.1093/braincomms/fcag224 (PMC13326950; doi:10.1093/braincomms/fcag224)
Supplement: fcag224_Supplementary_Data [file fcag224_supplementary_data.pdf]

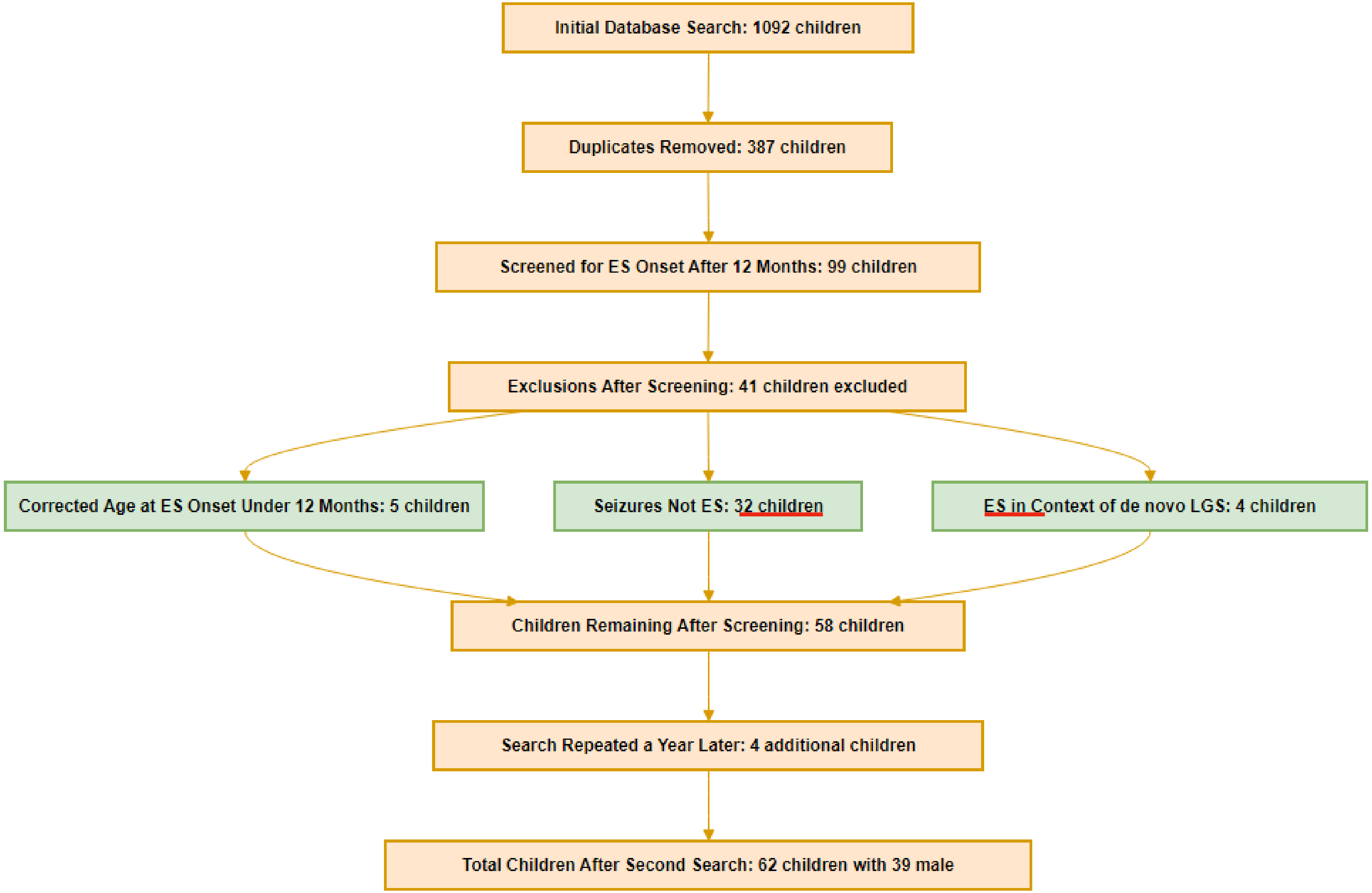

Supplementary Figure 1. Selection of participants for the late-onset epileptic spasms cohort.

Tree diagram showing selection of participants for the late-onset epileptic spasms cohort. The database search, screening and exclusion process was repeated 12 months later to capture potential delayed presentations.

Supplementary Table 1
Patient level data summary for 62 children with late-onset epileptic spasms.

Patient	Aetiology			Sex	Age at ES Onset (months)	Delay in ES Diagnosis (months)	Other Seizures Prior to ES	Delayed Development Prior to ES	ES Cessation With Medical Treatment*	Epilepsy Surgery	Seizure Outcome	Developmental Outcome	
	Category	Aetiology	Pathogenic Genetic Variant									Vineland ABC Score	WPPSI/ WISC category
1	Unk			M	20	36		N		No surgery	ES, FIAS	51	
2	S-A	[%]L PCA territory stroke		M	56	4	focal motor (ASS)	Y		No surgery	FIAS, ES, TCS, tonic (LGS)	38	Severe ID
3	S-M	[%]R frontal FCDII	^NVI (Brain)	F	14	13		N		Corticectomy	[#]Seizure free	normal cognition and schooling	
4	S-M	R frontal FCDII	NVI	M	63	26		N	VGB	No surgery	Seizure free	56	
5	Gen	*FOXG1* syndrome	*FOXG1*	F	24	14		Y		No surgery	tonic	26	Severe ID
6	Gen	Chromosomopathy	Unbalanced translocation of chromosomes 8 & 16	F	20	3		Y	PNL	No surgery	FIAS, TCS	24	Severe ID
7	Onc	acute lymphoblastic leukaemia		M	134	20		Y		No surgery	ES, tonic, TCS (LGS)	54	Severe ID
8	S-M	R frontal FCD		M	27	6		Y	TPM	No surgery	FIAS	51	
9	S-M	R hemisphere FCD		M	42	9		N		Lobectomy	TCS, ES	52	Moderate ID
10	S-A	Ureaplasma meningitis		M	13	1	focal motor, FBTCS (ASS)	N	VGB	No surgery	Seizure free	58	

11	Gen	Chromosomopathy	14q22.3q23.1 microdeletion (*OTX1*)	F	14	0		Y	PNL	No surgery	Seizure free	40	
12	S-A	L temporal-insula venous infarct		M	28	23	focal motor (ASS)	Y		Corticectomy	FIAS	55	
13	S-A	HSV meningitis		F	31	9	focal motor (ASS)	Y		Lobectomy	ES, tonic	44	Moderate ID
14	S-M	L frontal FCDII	*MTOR* (somatic)	M	30	0		Y		Corticectomy	Seizure free	85	
15	Gen	HECW2 related developmental disorder	*HECW2*	M	18	0		Y	CLB	No surgery	Seizure free	33	Severe ID
16	S-M	R frontal-temporal FCD	NVI	F	37	112	focal motor	N		No surgery	FIAS	54	Moderate ID
17	S-M	R frontal FCD		M	13	2		N	CLB	No surgery	Seizure free	88	
18	S-M	MOGHE	*SLC35A2* (somatic)	M	27	15		N		Corticectomy	ES, TCS	69	
19	S-M	lissencephaly	*LIS1*	F	23	1		Y		No surgery	ES, TCS, gelastic	severely delayed and special schooling	
20	S-M	tuberous sclerosis	*TSC1*	F	21	7		Y		No surgery	FIAS	50	Moderate ID
21	S-M	R frontal-temporal FCD		M	12	1	FBTCS	N	VGB	No surgery	Seizure free	54	Mild ID
22	S-M	tuberous sclerosis	*TSC1*	M	16	0		N		Tubectomy	FIAS	69	

23	S-A	R MCA territory stroke		M	14	0	focal motor (ASS)	Y	PNL	No surgery	Seizure free		Mild ID
24	S-M	L frontal-temporal FCD		M	15	1	FBTCS	Y	PNL	No surgery	Seizure free		Mild ID
25	S-M	L frontal FCDI	NVI (Brain)	F	18	44	focal motor	N		Corticecto my	Seizure free	84	
26	Gen	*PURA*- related neurodevelopmental disorder	*PURA*	M	13	0		Y	PNL	No surgery	Seizure free	36	
27	S-M	L frontal-temporal FCDI	NVI (Brain)	M	62	11		N	CLB	Lobectomy	Seizure free	78	
28	Unk		NVI	M	146	7		N		No surgery	TCS	48	Severe ID
29	S-M	L frontal FCD	NVI	F	30	0		N		No surgery	TCS, ES	53	Severe ID
30	Onc	acute myeloid leukaemia		F	45	1		Y	CBD	No surgery	Seizure free	57	Moderate ID
31	S-M	R frontal FCD		M	108	8		N	VGB	No surgery	Seizure free	normal cognition and schooling	
32	S-M	L frontal-insular FCD		F	31	12	FBTCS	N	LCM	No surgery	Seizure free	54	Mild ID
33	S-M	tuberous sclerosis	NVI	M	20	0	focal motor, FBTCS	Y	CLB	Tubectom y	FIAS,to nic	51	Moderate ID
34	S-M	R frontal FCDI	NVI (Brain)	M	18	52		N	CLB	Corticecto my	Seizure free	83	

35	S-M	L frontal FCDII	NVI (Brain)	M	35	9		Y	CLB	Corticectomy	FIAS	52	Moderate ID
36	S-M	tuberous sclerosis	NVI	M	27	2	focal motor	Y	VGB	No surgery	Seizure free		Mild ID
37	S-M	R frontal-temporal FCDI		M	12	0	focal motor	N		Hemispherotomy	Seizure free	72	
38	S-M	R frontal FCDI	NVI (Brain)	M	22	5	focal motor, FBTCS	N		Corticectomy	Seizure free	89	
39	S-M	subcortical band heterotopia	*DCX*	M	12	0		N		No surgery	ES, tonic, FIAS (LGS)	44	Severe ID
40	S-M	L frontal-insular-parietal FCD		M	13	0		Y	VGB	No surgery	Seizure free	69	
41	Onc	acute lymphoblastic leukaemia		F	180	3	focal motor, FBTCS (ASS)	Y		No surgery	TCS, ES, FIAS	51	Moderate ID
42	S-M	R frontal FCD	NVI	M	16	11		Y	CLB	No surgery	Seizure free	66	
43	S-M	R frontal FCD		M	31	6	focal motor	Y	CLB	No surgery	Seizure free	88	
44	S-M	L temporal-insular FCD		F	18	61	focal motor	Y		No surgery	FIAS	42	
45	S-A	L MCA territory stroke		M	36	17	FBTCS (ASS)	Y	VGB	Corticectomy	Seizure free		No ID
46	S-M	MOGHE	*SLC35A2* (somatic)	F	15	0		N		Lobectomy	FIAS	66	

47	S-M	R frontal FCDII		F	13	7		N		Corticectomy	Seizure free		No ID
48	S-M	tuberous sclerosis	*TSC2*	M	49	3		N	VGB	No surgery	Seizure free		Mild ID
49	S-M	R temporal ganglioglioma	*BRAF* (somatic)	M	20	6	focal motor	Y	PNL	Lobectomy	Seizure free	87	
50	S-A	L MCA territory stroke		M	23	0	status epilepticus (ASS)	Y		Hemispherotomy	Seizure free		No ID
51	S-M	tuberous sclerosis	*TSC2*	M	13	3		Y		Tubectomy	Seizure free	86	
52	S-M	subcortical band heterotopia	*DCX*	F	24	56		Y	VGB	No surgery	TCS, tonic, ES	54	
53	S-M	R frontal FCD		F	66	5		N	VGB	No surgery	Seizure free		Mild ID
54	S-M	MOGHE		M	17	21		N		Corticectomy	Seizure free	84	
55	Gen	*CACNA1AC*- related disorders	*CACNA1C*	M	16	0		Y	PNL	No surgery	Seizure free	severely delayed and special schooling	
56	Gen	*NRROS* -related neurodegenerative disorders	*NRROS*	F	13	2		Y		No surgery	FIAS, TCS, ES	severely delayed and special schooling	
57	Gen	Chromosomopathy	11q13.1-13.2 deletion	F	66	15		Y		No surgery	TCS, tonic, myoclonic (LGS)	34	Severe ID
58	S-M	R frontal FCD	NVI	M	19	10		N	VPA	No surgery	Seizure free		Mild ID

59	Unk		NVI	F	168	0		N		No surgery	TCS, FIAS, tonic (LGS)	42	Severe ID
60	Onc	acute lymphoblastic leukaemia		M	76	3		Y		No surgery	ES, TCS	50	
61	S-A	traumatic brain injury		F	129	13		Y		No surgery	ES, TCS, tonic, FIAS	severely delayed and special schooling	
62	S-M	R frontotemporal FCD	NVI	F	120	4	focal motor, FBTCS	Y		No surgery	ES, FIAS, tonic (LGS)	40	Moderate ID

S-M= structural malformative, S-A=strucural acquired, Gen=genetic, Onc= oncological, Unk= unknown
FCD= focal cortical dysplasia, PCA=posterior cerebral artery, HSV= Herpes simplex virus, MCA= middle cerebral artery, MOGHE= Mild malformation of cortical development with oligodendroglial hyperplasia and epilepsy

PNL= prednisolone, VGB= vigabatrin, TPM=topiramate, CLB=clobazam, VPA=valproate, LCM=lacosamide, CBD= cannabidiol

FIAS= focal impaired awareness seizure, FTBTCS = focal to bilateral tonic clonic seizure, ASS = acute symptomatic seizure, TCS=tonic clonic seizue, ES=epileptic spasms, LGS= Lennox-Gastaut syndrome
ABC= Adaptive Behaviour Composite, WPPSI=Wechsler Preschool and Primary Scale of Intelligence, WISC=Wechsler Intelligence Scale

* Response to medical treatment—whether as monotherapy or adjunctive therapy—was defined as cessation of epileptic spasms sustained for a minimum duration of 12 months
